# Information-Seeking at a Caregiving Website: A Qualitative Analysis

**DOI:** 10.2196/jmir.1548

**Published:** 2010-07-28

**Authors:** Leslie P Kernisan, Rebecca L Sudore, Sara J Knight

**Affiliations:** ^2^Interdisciplinary Program to Improve Care for Veterans with Complex Comorbid ConditionsSan Francisco VA Medical CenterSan FranciscoUnited States; ^1^Division of GeriatricsUniversity of California San FranciscoSan FranciscoUnited States

**Keywords:** Caregivers, Internet, consumer health information

## Abstract

**Background:**

The Internet is widely used for health information, yet little is known about the online activity of family caregivers of elders, a rapidly growing group. In order to better understand the online information-seeking activity of “e-caregivers” and other visitors at a caregiving website, we undertook a qualitative analysis of survey data from a website marketed as a comprehensive resource for adults caring for aging parents.

**Objective:**

The objectives were to better understand what types of information are sought by those visiting a website focused on elder-care issues and to identify overarching themes that might inform future development of Internet resources related to caregiving and aging.

**Methods:**

From March 2008 to March 2009, a 5-question pop-up survey was offered 9662 times and completed 2161 times. For 1838 respondents, included was a free text answer to the question "What were you looking for?” and 1467 offered relevant and detailed responses. The survey also asked about satisfaction with the site, gender of the respondent, and relationship to the individual being cared for. Content analysis was used to develop a coding dictionary, to code responses into information-seeking categories, and to identify overarching themes.

**Results:**

Of the respondents (76% of whom were female), 50% indicated they were caring for parents, 17% for themselves only, and 31% for others. Over half (57%) reported finding what they were looking for, and 46% stated they were extremely likely to recommend the website. Frequently mentioned information-seeking categories included “health information,” “practical caregiving,” and “support.” Respondents also requested information related to housing, legal, insurance, and financial issues. Many responses referred to multiple comorbid conditions and complex caregiving situations. Overarching themes included (1) a desire for assistance with a wide range of practical skills and information and (2) help interpreting symptoms and behavior, such as knowing what life impacts to expect over the course of a health condition or treatment.

**Conclusion:**

Visitors to a website targeting adults caring for aging parents reported seeking both general information on caregiving and specific assistance with the complex custodial, medical, emotional, and financial aspects of caregiving. Visitors requested both information to build caregiving skills as well as assistance in interpreting and knowing what to expect from symptoms, health conditions, and changes in behavior and relationships. Many desired communication with and support from other caregivers. Health care providers and eHealth developers should expect that many caregivers of elders are using the Internet as a resource. Further research and development is needed to fully realize the Internet’s potential for education and support of caregivers.

## Introduction

While the Internet is one of the most widely used resources for health information [1,2], its role in providing consumer information specifically related to the health care and living needs of frail elders (and other dependent adults) remains less well understood. Relatively few older Americans use the Internet regularly [3]. One 2004 survey found that only 31% of respondents older than 65 years had ever gone online, and only 8% said they obtained “a lot” of health information online [4]. Nonetheless, in recent years websites providing elder-focused consumer health information have become more common [5]. This may be partly due to a realization that, although the elderly are often not themselves online users, their needs for care may prompt their caregivers to search the Internet for information [6]. For instance, a recent Pew report found that about half of all online health inquiries are on the behalf of another person [1].

The number of elderly people in America is rapidly growing, with the number of those aged 85 and over projected to more than triple by 2040 [7]. However, as noted in *Retooling for an Aging America*, a 2008 Institute of Medicine (IOM) report [8], the nation is not well prepared to meet the complex medical and social needs of a growing frail population. To address this pressing public health issue, the IOM’s key findings included a recommendation that the United States “better prepare informal caregivers to tend to the needs of aging family members and friends” [9]. Currently, an estimated 30 to 40 million Americans provide unpaid care to relatives and friends, the value of which has been estimated at $375 billion [10-13]. Caregivers are often integral to facilitating medical and personal care for frail elders [11,14,15]. Yet, this “shadow workforce in the geriatric care system” continues to feel inadequately informed, prepared, and supported [16]. Societal trends indicate that use of the Internet to meet information needs will almost certainly continue to increase. It is clear that developing a better understanding of “e-caregivers” will be necessary if the resources and capabilities of eHealth are to be properly leveraged to best serve the elderly, the disabled, and the millions who care for them.

Although other scholars have used the term “e-patient” to refer to “both those who seek online guidance for their own ailments and the friends and family members who go online on their behalf” [17], in this paper we will use the term “e-caregiver” to refer to those online seeking guidance related to helping another person with their medical care or personal care.

To date, little has been published regarding who these e-caregivers are and what they are seeking to learn or obtain through the Internet. While some empirical evidence exists that documents caregivers’ use of Internet health care resources, especially for specific conditions such as cancer and stroke [18-20], the online information seeking of caregivers in the broader context of frail and vulnerable adults has hardly been studied. In particular, although two studies used caregiver focus groups to elicit suggestions regarding potential eHealth applications [21,22], this work has not yet been followed by more detailed observational analyses of what caregivers may be actually seeking when they turn to the Internet, which must be better understood if eHealth resources are to be properly developed and refined.

The existing literature on caregiving does describe many facets of the preInternet caregiver experience, and this work could be used to inform the expansion of caregiver studies to the realm of eHealth. Frameworks of caregiver needs have been proposed, almost always tailored to the context of specific diseases and conditions. For instance, in a study of information and service needs in dementia, Edelman et al organized caregiver (and care recipient) concerns into 4 main domains: care, coping, medical, and services [23]. Other authors have roughly categorized caregiver needs as being informational (about illness, services, and what to expect), instrumental (related to building caregiving skills), and emotional (related to support and coping) [24-26]. While these conceptual frameworks can provide a foundation for understanding the needs of caregivers who seek information online, they are limited in two important respects. First, they were not developed from the perspective of caregiving for the frail elder, a context in which multiple complex medical and psychosocial conditions (including the possibility of dementia prior to a clear diagnosis) are the norm, as are evolving caregiver responsibilities. Second, these frameworks predate the dominance of the Internet for information seeking, which is producing significant cultural shifts in information behavior and health behavior [27] and, hence, might influence the types of caregivers who seek help and the domains of needs that are expressed.

To help address these gaps in the understanding of e-caregivers and their needs, we conducted a qualitative analysis of over 2000 responses to a Web-based survey conducted in 2008 and 2009 as part of routine research and development at Caring.com, a website created to provide support and informational resources to adults caring for aging parents. Our goal was to understand what types of information were being sought, especially to explore whether new domains should be considered for the existing conceptual models. Through qualitative analysis, we also sought to explore overarching themes that cut across the specific information-seeking categories.

## Methods

### Study Design and Sample

Our study consisted of a secondary analysis of responses to a five-question Web-based survey. Data were obtained from Caring.com, a website launched in November 2007 and designed to function as a comprehensive resource for adults caring for aging parents. The website provides informational articles about common medical problems affecting seniors, articles on caregiver well-being and managing difficult family dynamics, information on housing options for elders, as well as blogs, discussion forums, and postings of answers by experts in health and eldercare to visitors’ questions. To conceive of the major sections of the website, the founders relied both on their own personal experiences struggling to help aging parents, as well as on their professional experience working with Babycenter.com, a successful and well-established website for expectant parents and people with small children. The vast majority of Caring.com’s content is original and written by the company’s health writers, and users are not required to pay any fees (although free registration with the site is encouraged, and sometimes required to access certain features). During the study period, Caring.com was supported by venture capital funds, with a plan for the company to eventually support itself through revenue from those wishing to advertise eldercare services and supplies. Caring.com’s editorial policy forbids advertisers from participating in the creation of the written content.

The survey had been implemented for the purpose of quality improvement and was available via Caring.com’s website for 12 calendar months, beginning in March 2008. The survey was offered to users of the site via a pop-up invitation to help improve Caring.com. Those who agreed to participate were redirected to a Zoomerang survey that presented five items, four with multiple choice response sets, and one open-ended question. The specific questions were: (1) How likely is that you would recommend Caring.com to a friend or relative? (scored 0 to 10 where 10 = extremely likely) (2) What were you looking for when you came to the Caring.com website? (3) Did you find what you were looking for? (4) Who are you caring for? and (5) What is your gender? For the open-ended question (What were you looking for?), the survey provided space for unlimited free text responses. A more detailed description of the survey, in accordance with the checklist for reporting results of Internet e-surveys (CHERRIES) guidelines [28], is provided in Multimedia Appendix 1.

We collected all survey data available from the time of the survey’s launch in March 2008 (approximately five months following the website’s launch) until the website closed the survey in March 2009. At the time the survey became live, the website was attracting approximately 6500 visits per week. By March 2009, the site traffic had increased to approximately 75,000 visits per week. At this time, the survey had been viewed 9662 times and was submitted 2161 times (22.4% participation rate), of which 1838 responses included an answer to the free-text question “What were you looking for?”

The raw data were imported into Microsoft Excel 2007 (Microsoft, Redmond, WA) and into StataMP version 10.0 (StataCorp, College Station, TX), which we used to compile descriptive statistics. Use of the data for this project was approved as exempt by the University of California, San Francisco Committee on Human Research, and by the San Francisco Veterans Affairs (VA) Medical Center Committee on Research and Development.

### Respondent Characteristics

Descriptive statistics were used to describe those respondents included and excluded from content analysis of the free-text question. We also tabulated the survey results by response to the question “Who are you caring for?” Respondents had indicated one or more of the following choices: parent(s), grandparent(s), spouse, sibling(s), self, other older loved one(s), and other. This resulted in 69 different combinations of care recipients, and hence, these answers were recoded into the following 4 respondent types: those caring for parents (those who indicated parent(s), whether or not any other care recipient was also indicated); those caring for self only; all other caregiving situations; and unknown (for those who did not choose any of the options).

### Analysis of Free Text Responses

The 1838 free-text responses to the question “What were you looking for?” were analyzed using content coding. Initially, all free text answers were reviewed by one of the investigators (LK) to identify themes and patterns among the responses and to construct a preliminary coding manual. Based on the preliminary review of the data, we excluded 371 respondents from further analysis because their free-text answers were irrelevant to the purpose of the website (eg, “weather”), indicated an unintentional visit to the site (ie, “got here by accident,” or “nothing”), or were too nonspecific to be categorized (eg, “an article” or “not sure”). This left us with 1467 free-text responses to further classify into information-seeking categories. Although we initially considered excluding responses from those who reported caring for themselves only, we chose to include them in our analysis as we felt there was value in understanding what information a noncaregiver might be seeking at a caregiving website.

We refined the coding manual by having two investigators (LK and SK) apply the preliminary category codes to a sample of data (1000 responses) followed by discussion and further refinement of the categories and their criteria. Table 1 shows the final categories and the defining criteria for each. The nine categories were: health information, practical caregiving, behavioral, support, legal/financial/insurance, housing/living situations, driving, unspecified need for help/information, and “other.” The “other” category was used to capture responses that were explicit and specific but unique and not able to be grouped within another category.

**Table 1:** Information-seeking category codes based on content analysis

Category	Free-Text Reponses Indicated Respondent Looking for:	Examples
Health information	Information about diseases, medical condition, health care	"Dementia," "health tips"
Practical caregiving	Information about how to provide care to another person	"Tips on bathing," "Alzheimer's care"
Help/information, nonspecific	Information and/or help, without further specification	"Info to help my aging parents"
Legal/financial/insurance	Information about legal matters, financial issues, or health insurance	"Long-term care insurance," "estate tax info"
Behavioral	Help addressing behavioral, psychological, and relationship issues	"Ways to understand what they say without causing an argument"
Support	Emotional support from other site participants and/or help coping with stress	"Someone to relate to," "support group and answers"
Housing/living situations	Information about housing, placement, parent moving in	"Nursing home," "whether my mother should move in"
Driving	Information about elders and driving	"How to take the keys away"
Other	Specific information that doesn't fall into any other domain	"Emergency pendants," "how to write a eulogy"
Excluded	Text shows only vague interest, is uninterpretable, or seems not at all relevant to site	"Just looking," "opinions," "the weather"

Following the development of refined codes, both investigators classified the content of the 1467 free text answers according to the primary category represented in the text. An initial assessment of interrater agreement for the two investigators was 83% indicating good interrater reliability. To generate the final counts within each category, coding discrepancies were resolved through discussion with a third researcher (RS). The category counts were summarized by caregiver type (ie, parent, self, all other, and unknown).

In a final step, we selected four of the frequently coded categories where the responses were of sufficient length to allow further interpretation. These responses were reviewed, using inductive content analysis [29], to provide a deeper and more nuanced understanding of the responses and to identify overarching themes that occurred across all of the categories.

## Results

### Respondent Characteristics

Of the 2161 submitted surveys, 1838 (85%) provided free-text answers to the question “What were you looking for?” Of the free-text answers, 1467 of 1838 (80%) contained sufficient detail relevant to caregiving to be considered for interpretation and thus inclusion in the content analysis. Characteristics of the respondents included in the content analysis, compared with those not included, are presented in Table 2. Excluded respondents were more likely to be male, were less likely to be caring for parents, were less likely to report finding what they were looking for, and were less likely to say that they would recommend the site to others.

**Table 2:** Respondent characteristics

		Included^a^		Excluded^a^		Total	
Respondent Characteristic/Response		n = 1467		n = 694		n= 2161	
		n	%	n	%	n	%
**Gender**							
	Female	1,115	76%	462	67%	1,577	73%
	Male	339	23%	201	29%	540	25%
	Missing	13	1%	31	4%	44	2%
**Who caring for**							
	Parent(s) or parent(s) and other(s)	739	50%	246	35%	985	46%
	Self only	249	17%	162	23%	411	19%
	Other caregiving situations	450	31%	236	34%	686	32%
	Missing	29	2%	50	7%	79	4%
**Information found?**							
	Yes	831	57%	302	44%	1,133	52%
	No	91	6%	53	8%	144	7%
	Unsure	538	37%	301	43%	839	39%
	Missing	7	0%	38	5%	45	2%
							
**Mean recommendation score**		8.1		7.0		7.7	
	95% confidence interval (CI)	7.9 - 8.2		6.8 - 7.3		7.6 - 7.8	

^a^ Included in or excluded from the content analysis (included if respondent provided a sufficiently detailed and relevant response to "What were you looking for?")

### Who Respondents Reported They Were Caring For

Respondent characteristics varied somewhat by who the respondent reported caring for, as shown in Table 3. Those caring for parents were more likely to be female and to have indicated that they had found the information they were looking for on the site. This group also had the highest mean score on the scale indicating likelihood of recommending the site to others.

**Table 3:** Respondent characteristics by whom they reported caring for, for content analysis sample

		Caring for Parents	Caring for Self Only	Other Caregiving Situation	Unknown Caregiving Situation	All
Characteristic/Response		n = 739	n = 249	n = 450	n = 29	n = 1467
		% of n	% of n	% of n	% of n	% of n
**Gender**							
	Female	79.6%	67.5%	75.6%	65.5%	76.0%
	Male	19.6%	31.7%	24.0%	24.1%	23.1%
	Missing	0.8%	0.8%	0.4%	10.3%	0.9%
**Information found?**							
	Yes	60.0%	51.8%	54.2%	51.7%	56.7%
	No	3.4%	11.7%	7.8%	6.9%	6.2%
	Unsure	36.3%	36.1%	37.6%	37.9%	36.7%
	Missing	0.4%	0.4%	0.4%	3.5%	0.5%
						
**Mean recommendation score**		8.4	7.6	7.9	6.7	8.1
	95% CI	8.2 - 8.5	7.3 - 8.0	7.7 - 8.2	5.4 - 8.0	7.9 - 8.2

### Information-Seeking Categories

Responses to the question “What were you looking for?” were coded into 9 specific categories through content analysis. Frequency counts of information-seeking domains (by who the respondent reported caring for) are presented in Table 4. Among those caring for parents, “practical caregiving” was the most frequently coded domain.

**Table 4:** Information-seeking categories by whom respondent reported caring for

	Caring for Parents	Caring for Self Only	Other Caregiving Situation	Unknown Caregiving Situation	All
Information-Seeking Category	n = 739	n = 249	n = 450	n = 29	n = 1467
	% of n	% of n	% of n	% of n	% of n
Health information	18%	46%	36%	31%	29%
Practical caregiving	32%	6%	13%	14%	21%
Help/information, nonspecific	16%	16%	16%	10%	16%
Legal/financial/insurance	9%	13%	11%	17%	10%
Behavioral	6%	7%	7%	10%	7%
Support	10%	3%	5%	0%	7%
Other	4%	3%	8%	10%	5%
Housing/living situations	3%	4%	2%	0%	3%
Driving	2%	2%	2%	7%	2%

### Qualitative Findings

Qualitative findings from the content analysis of four frequently coded categories (health information, practical caregiving, behavioral, and support) are presented below. Although “help/information, non-specific” was relatively frequently coded, the brief responses within this domain were not suitable for further qualitative analysis. Through our qualitative analysis, we also identified two overarching themes that cut across most of the information-seeking categories.

### Health Information

A number of respondents requested information about conditions, diseases, and aging. Diseases and conditions mentioned included cancer and cancer treatment side effects, dementias, diabetes, heart disease, and stroke. A few responses specified a need for information regarding geriatric health, or senior health.

Many respondents asked about signs and symptoms of disease, especially as related to dementia and cancer, for example, “signs of Alzheimer’s” and “if low red blood cell tests could be an indicator to cancer.” Other respondents posed narrowly focused questions about symptoms, such as whether there is a “different sound a person makes at the final stage of the disease,” and whether a person can “be sensitive to touch when they have Alzheimer’s?”

There was also interest in the treatment and management of health conditions such as anemia, dementia, and diabetes, suggesting that respondents were engaged in the monitoring and management of either their own health, or that of another. Some responses reflected an urgent need for information (eg, “time limit on an open insulin bottle,” “antidote to overdose of tick medication for my wife,” and “how to care for raw burn from breast radiation.”) Relatively few responses specifically asked about managing pain or other symptoms, and few asked about “cure,” although many responses cited chemotherapy or other therapies that are often delivered with curative intent. In spite of the interest in treatment of disease, there were few responses that mentioned an interest in how to interface with health providers or navigate the medical system.

Questions related to what to expect or anticipate in the future emerged repeatedly within this domain, for example, “How long will chemo stay in the body?” There was a particular interest in the course of dementia, suggesting a need for information to plan for the future or possibly to aid in decision-making, for example, “How long will an Alzheimer’s patient live after being diagnosed?”

Some responses indicated an interest in the prevention of health problems such as heart attacks, diabetes, and dementia. Others asked for tips on staying fit physically or mentally. We also found that some requests indicated a desire for causal explanations and greater understanding of health problems. For instance, respondents asked for explanations of Alzheimer’s, the cause of a parent’s memory loss, and the causes of low blood cell counts.

### Practical Caregiving

Many respondents indicated that they were looking for specific advice on the practical aspects of managing the daily living needs of another person, with a majority of these making reference to caring for parents with dementia or other frailty, for example, bathing, giving a pedicure, using a gait belt, hiring care workers, finding affordable services, and getting respite care. Knowing what to expect and how to plan for caregiving emerged as central needs. For example:

My parents are at the age that we need to hire in home care. Their health is failing and my family needs more information about changes in health, what to expect, and how to plan.

…some insight as to what my mother is going through and what to expect so I could take care of her better

A number of the caregiving responses reflected concerns about understanding the needs of the person receiving care and a desire to improve life for this person (eg, “searching for activities for my extremely nearsighted mother”). These questions focused on concern about reducing discomfort and pain, finding enjoyable activities, and respecting the wishes of the person receiving care. Some answers also indicated that respondents were involved from a distance or were trying to help another caregiver:

I was hoping to find something that would help me figure out a solution for a distant relative who lives alone

…information for my mother who is caring for my grandparents—who are in their 90s, living at home, and impossible

A number of respondents asked questions about new caregiving situations for which decisions were required, for example, following a discharge from the hospital or sudden increase in caregiving responsibilities with functional decline. Similarly, a number of questions focused on caring for multiple disabled or frail parents or how to provide personal care to a resistant care recipient. Finally, respondents asked about how to balance the challenges of caregiving with other life activities, for example, work and caring for children. Several described being a caregiver while coping with one’s own illness or disability.

### Behavioral

Many respondents indicated that they were looking for assistance with behavioral concerns. These responses touched on communication concerns, relationship issues, psychological concerns, as well as management of behavioral symptoms and sleep difficulties.

Responses within this domain often alluded to struggles to understand and cope with relationships challenged by illness and aging. Several respondents indicated a desire to better understand a parent or other person or asked how to raise difficult topics for discussion:

Insight on my elderly parents actions

Ways to understand what they say without causing an argument

Help on talking to my child about my parent's death

What issues that could arise between you and your spouse's parents, and how it may positively and negatively affect the marriage

Why do adult children turn away from their elderly parents?

Respondents frequently indicated a need for assistance with the behavior of a loved one in the context of dementia. For instance, several responses indicated a need for information on how to care for a parent who is having difficulty accepting loss of independence with early dementia or how to communicate with someone who has cognitive impairment and emotional instability.

Ideas for how to get an intransigent 88-year-old Alzheimers patient (my dad) to accept the help he desperately needs but refuses because he does not want to spend the money and cannot accept our role reversals

How to talk with a mother who is probably in the [second] stage of Alzheimer’s and is not always logical, has mood swings, [and is] extremely forgetful

Suggestions for communicating with parents in early stages of Alzheimer's

Other responses indicated an interest in information relating to common neuro-psychiatric syndromes, such as sundowning, hallucinations, and hoarding. For example, respondents asked for “description of sundown dementia” or for “a simple, easy-to-understand definition between a hoarder and a pack rat.” These responses suggested that respondents may be attempting to interpret and explain the behaviors they are observing. One particular response indicated a search for an explanation for “erratic behavior,” suggesting a diagnosis still in question. Several responses requested explanations and tips for addressing sleep difficulties.

### Support

Many responses indicated that a primary goal of the respondent was to find sources of emotional support through the website. Most respondents seemed particularly interested in the experiences of others in similar situations, and some specified a desire to know that others were having similar feelings and struggles.

…to know I’m not the only one in the world going through this

…others caring for terminal parent

My mother has Alzheimer’s. My heart breaks. I wanted to see how others are coping.

Several respondents also expressed a desire to exchange ideas with peers, for example, they asked for a “sounding board” or “ways to ask questions of others going through the same thing.” Furthermore, after expressing a desire for “advice, experience of other people,” one respondent continued by adding, “more importantly I was looking for authentic information, not just ‘Dear Abby’ style answers to broad questions.”

A number of respondents expressed a desire to feel less alone and referred to feeling stressed and overwhelmed. Several noted a need for comfort and relief, and many asked for information on how to better cope. A few respondents did not explicitly seem interested in a community of peers online, but rather asked for broader information on caregiver support resources. One respondent expressed a need for support in being a care recipient rather than a caregiver.

…something other than pat answers and harsh judgment…I often feel disappeared by the very people who supposedly care for me. They make decisions about me without including me.

Many responses indicated that respondents were interested in using the Internet to connect with other caregivers. Through these connections, respondents indicated interests in exchanging ideas, normalizing their experiences, and feeling that they are part of a community.

### Overarching Themes Identified Through Qualitative Analysis

Two overarching themes emerged from the qualitative analysis that were pertinent to and spanned all 9 content categories: (1) a desire for assistance with a wide range of practical skills and information and (2) a search for help interpreting symptoms, behaviors, and interpersonal situations, and in knowing what to expect and how to plan.

### Practical Skills and Information

Many respondents indicated that they were looking for practical skills and information to address a current situation, especially in the context of providing or facilitating care for another person. In other words, respondents often asked for information on how to do something. For instance, within the “practical caregiving” category, respondents requested information on how to hire a home care worker and how to select a personal medical alarm. A desire for practical skills and solutions was also evident in responses coded into the “health information,” “housing/living situations,” “legal/financial/insurance,” and “driving” categories. For instance, respondents asked for information on how to choose nursing homes and continuing care retirement facilities and how to establish trusts and wills. Another respondent asked “how to take the parents’ car keys when they refuse.”

Respondents also often requested practical skills related to communication and managing relationships, especially in the context of dementia or the discussion of difficult topics.

How to deal with a parent with dementia…different strategies to talk to doctor and respond to unusual behavior

Advice on how to cope without getting exhausted

Help with moving our parents into an assisted living home and making it their idea

### Help Interpreting Situations and Knowing What to Expect

Another theme that emerged across the categories was the need for help in interpreting situations, often so as to know what to expect and, thus, what to do (as opposed to how to do things). Responses illustrating this theme were found within virtually all content categories but were particularly common within “health information,” “behavioral,” “support,” as well as “practical caregiving.”

My parents are at the age that we need to hire in home care. Their health is failing and my family needs more information about changes in health, what to expect, and how to plan.

…what to expect with the end stage of CHF [congestive heart failure]

…to see if what I felt and am doing is of the norm

Within the “housing/living situations” category, respondents asked for help knowing when it might be time for an older person to be moved from their home. Within “driving,” they requested guidelines to know when driving should be stopped.

## Discussion

### Principal Results

Through our qualitative analysis of 1467 text responses about what people were looking for at a general caregiving website for aging parents, our study provided a clear view of how some e-caregivers are seeking to meet their needs through the Internet. Among our sample, “health information” and “practical caregiving” were the two most prevalent categories of interest; other frequently mentioned topics encompassed legal/financial/insurance issues, support issues, behavioral issues, and housing/living situations. Many of the needs expressed were not specific to a particular medical condition. There was considerable interest in help with knowing what to expect and in how to plan for the future. There were also many concerns related to understanding behavior and relationships, as well as interest in support and assistance with coping. Many respondents expressed an interest in communicating with other e-caregivers. Furthermore, we noted that several geriatric topics appeared to be prominent concerns for these e-caregivers, including functional decline, cognitive impairment, and challenges with independent living.

### Comparison With Prior Work

Our findings are consistent with previous research on caregivers, which has suggested that caregivers’ needs include needs for information on diagnosis and prognosis, for practical information on implementing caregiving, and for support and assistance in coping. While we observed that visitors to a caregiving website spontaneously raised concerns that were similar to those documented in nononline settings [23-26], our qualitative findings highlight the breadth and depth of information needs that might be brought to an eHealth resource focused on aging and caregiving issues. For example, beyond wanting to know about prognosis, respondents expressed a desire to understand how to anticipate and plan for the impacts of another’s declining health across a wide range of domains of living. These results attest to the fact that caring for a dependent adult can generate a dizzying array of questions, which caregivers are now bringing to the Internet.

Our study also provides a unique perspective on what these e-caregivers may be less concerned about finding online. Given the fairly large sample size, we were struck by the relative paucity, or even frank lack, of mention of certain topics that frequently are found in the scientific and professional literature on elders. For instance, although several respondents expressed interest in symptom interpretation, few responses pertained to the treatment of pain or other uncomfortable symptoms, although pain is a common symptom in later life [30]. We also noted that only two responses specifically alluded to depression, although many referred to stress and sadness affecting caregivers and care recipients. Another omission we found conspicuous was a total absence of requests for information on advance care planning. (ie, living wills or advance directives). We furthermore noticed that no respondent explicitly linked concerns about driving to concerns about cognitive impairment. Among health professionals, these topics are widely believed to be relevant to a majority of frail patients. Experts also generally agree that these issues are usually insufficiently addressed in routine clinical encounters. That these issues should also be scarcely mentioned by visitors to a caregiving website raises the possibility that there is low awareness among a group that has been motivated enough to have visited a caregiving website and to have participated in a survey. This is in stark contrast to issues such as what to expect in the future, practical caregiver information, caregiver support needs, and behavioral issues, all represented within these survey results, but which are often inadequately addressed in clinical encounters. However, it is also possible that the omissions we noted do not reflect low awareness, but instead are due to factors such as website traffic or perhaps respondents’ experience at the website (ie, the site may have not promoted its advance care planning information as frequently as its driving information).

### Limitations

There are some limitations that are important to consider in the interpretation of our results. The first and main one is that our sample was drawn from one particular Internet site. Consequently, our frequency counts cannot be extrapolated to Internet-using caregivers in general. Our sample was also likely influenced by factors related to Caring.com itself, a site which has marketed itself broadly to boomers, and through mainstream Internet health portals (ie, AOL, Yahoo Health, etc). Visitors can arrive at a commercial health information site such as Caring.com in many different ways, such as through a search engine or from a portal if they follow the link to an appealing-sounding article. Hence, although our respondents were visiting a caregiving website, and many identified themselves as caregivers, we cannot assume that all of them came to Caring.com specifically because it was a site with information related to caregivers. It is also possible that for some respondents, their expressed information needs may have been influenced by a perception of Caring.com as a certain kind of commercial site, as opposed to a government site, or site hosted by a medical specialty group. The process by which consumers arrive at websites and evaluate credibility and value is complex [31-36], so it is difficult to know just how these factors may have affected our sample. Furthermore, while the survey was presented at random to users during the data collection period, a nonrandom proportion was willing to complete the survey, and we have no data on those who refused to participate. Still, the number of responses that could be interpreted was large, and our participation rate of 22% is within one standard deviation of the mean Internet survey participation rate of 34% observed in a meta-analysis of 39 Internet surveys [37]. Thus, it is unclear how the information needs of the respondents would be biased beyond an interest in caregiving and senior health plus what would be expected based on what is already known about adults who use the Internet for health information [38,39].

Although our sample was likely influenced by a number of unknowable factors, our qualitative findings still provide an important complement to previous research conducted in settings other than online settings and have substantial value for generating hypotheses about e-caregiver information needs, which can be tested in future research. Some studies have examined caregivers’ use of Internet health care resources, yet this work, similar to much of the earlier research on caregivers, has mainly been focused on a single disease or health condition, such as stroke or dementia [20,40]. In contrast, our work identified several domains of caregiver needs that may cut across disease boundaries (eg, driving, housing, and financial issues), and explored caregiving questions related to complex situations involving several health conditions or psychosocial situations. As most elder care occurs within a dynamic context of multi-morbidity and functional impairment, the previous literature’s emphasis on specific health conditions poses a challenge for generalist providers of care to elders and their caregivers. In contrast to the prevalent disease-based approach, our findings will be highly relevant to the work of practicing primary care providers, eHealth developers, and others who must serve a diverse group of aging adults and informal caregivers.

An additional consideration important to note is that open health-related Internet sites, such as Caring.com, may attract a mixture of user types, some explicitly targeted by the site and others not targeted for whom some of the site material is relevant. In our study, the interpretable responses included 294 of 1467 (17%) from users who reported caring for themselves only, and not for another person. While Caring.com targets those who are caring for others, self-caring users are clearly a sizable minority of site visitors. Because these individuals seem to have considered themselves to be engaged in a type of caregiving and were interested enough to complete the survey while visiting the site, we reasoned that including these respondents in the qualitative analysis could inform the conceptualization of potential site users and expand the conceptual framework for development of online information materials on caregiving. The qualitative categorization of what these individuals were looking for in contrast to those taking care of others has value in developing hypotheses for future research about the possible differences and similarities in needs across a range of users. Future studies of those using caregiving websites may also benefit from a survey structure designed to distinguish between care recipients, who presumably will have information needs related to many caregiving topics, and individuals who are neither caregivers nor care recipients. Also, it is possible that some users who are neither caregivers nor care recipients may still be in search of information related to caregiving situations, perhaps in anticipation of future care needs. Future research will be needed to better understand how open websites related to caregiving can more effectively serve a range of users.

### Conclusions

For clinicians, our qualitative findings clearly emphasize the importance of providing prognostic and other anticipatory information to caregivers to facilitate planning for the future. Our results also confirm that there is a need for decision-making resources designed for those with caregiving concerns and that the Internet might become a very valuable medium through which caregivers can access helpful information to make decisions related to their loved ones. A recent Pew survey underscores the central role of Internet information in health planning and decision making. Of patients seeking health information online, 60% reported that online information affected a decision about how to treat an illness or a condition [1]. Our findings suggest that clinicians should expect that caregivers might be influenced as well by online information.

For eHealth researchers and developers, our study findings highlight the dynamic and complex information needs of caregivers and those visiting a caregiving website and suggest that e-caregivers may have needs that warrant them being considered as a group distinct from e-patients (rather than considered a subset of e-patients). Our findings also shed light on issues that are particularly relevant to applying eHealth to the care of elders. Although our respondents often expressed concerns that transcended the disease-focused categories around which many eHealth resources are organized, we found that much of the information sought by these e-caregivers corresponded to traditional areas of geriatric expertise, such as functional decline, cognitive impairment, and family dynamics stressed by uncertainties and an aging person’s increased dependence. To accommodate the complex needs of elders, the practice of geriatrics often focuses on syndromes, that is, clinical conditions in the elderly that usually have multi-factorial origins [41]. Common geriatric syndromes that a caregiver might seek assistance with include falls, dizziness, delirium, incontinence, and frailty; all can have significant impacts on quality of life as well as mortality and morbidity. Because these syndromes span diseases, organ systems, and age-related physical changes, they are often difficult to manage in the disease-based fashion that might work well in a younger person. Instead, geriatrics offers alternative clinical approaches, often interdisciplinary, to managing these very common problems that affect the elderly. Our study findings suggest that such a geriatric approach could be a valuable contribution to eHealth resources designed for elders, and those who care for them.

Given the aging of the population and the broad consensus that family caregivers are an essential component of our nation’s care system for the elderly, our results suggest that eHealth resources have great potential to reach and support this important population. To help develop this potential, more data will be needed to better characterize the e-caregiver population. For instance, it would be very useful for eHealth surveys, such as those conducted by the Pew Internet & American Life Project and the National Center for Health Statistics, to begin collecting data on e-caregivers and their behaviors. Future research should focus on developing effective eHealth resources to educate and support e-caregivers, and developers should consider incorporating geriatric principles into the organization of these resources.

## Multimedia Appendix 1

CHERRIES summary

## Abbreviations

CHF: congestive heart failure
CHERRIES: checklist for reporting results of Internet e-surveys
IOM: Institute of Medicine
VA: Veteran’s Affairs
