# Supplementary material for: Information-Seeking at a Caregiving Website: A Qualitative Analysis
Source: J Med Internet Res. 2010 Jul 28;12(3):e31. doi: 10.2196/jmir.1548 (PMC2956334; doi:10.2196/jmir.1548)
Supplement: Supplementary file 1 [file jmir_v12i3e31_app1.pdf]

Multimedia Appendix 1
CHERRIES Summary

| | | |
|---|---|---|
| **Design** | Survey Design | This was a Zoomerang survey created by a marketing research consultant, in conjunction with Caring.com's editorial and business management team. The authors of this research study were not involved in the design of the survey.<br>The purpose of the survey was for Caring.com to better understand who was visiting the site, and what was being looked for.<br>The target population was online visitors to Caring.com. |
| **IRB approval and informed consent process** | IRB Approval | Caring.com's development and use of the survey did not require IRB involvement, as the survey was developed internally for the company's research and quality improvement needs.<br>Use of the survey data for the purpose of an academic secondary data analysis was approved by the UCSF Committee on Human Research, and by the San Francisco VA Committee on Research and Development. |
| | Informed Consent | Participants were invited to participate in the survey through a pop-up inviting them to "Tell us what you think," in order to improve Caring.com. No other process for informed consent was used. No personally identifying data was requested. |
| | Data Protection | Prior to the commencement of our scholarly analysis, all data was collected and stored on Zoomerang's website.<br>For this study, the data (none of which included personal identifying information) was downloaded to the first author's UCSF laptop, which is encrypted, password-protected, and otherwise meets UCSF's standards for storing de-identified research data. |
| **Development /pre-testing** | Development and Testing | The survey was developed by Caring.com's marketing research consultant, in conjunction with the editorial and business management team. No formal pre-testing was undertaken. Survey development occurred prior to any study author's involvement with Caring.com. |
| **Recruitment process and description of the sample** | Open vs Closed | This was an open survey, accessed through the site visitor assenting to a pop-up invitation. |
| | Contact Mode | The initial contact with potential participants was made only through the pop-up invitation. The pop-up was programmed to appear during 5% of visits, after a visitor had been on the site for 120 seconds. |
| | Advertising | No advertising other than the pop-up invitation was used. |

| | | |
|---|---|---|
| Survey Administration | Web/Email | Once a potential respondent had indicated a willingness to participate in the survey (through the pop-up invitation), the respondent was immediately directed to a new window or browser tab, which allowed online completion of the survey on Zoomerang's website. No manual entry of responses was needed. |
| | Context | Caring.com is a for-profit informational website designed to function as a comprehensive resource for adults caring for aging parents. The site provides informational articles about common medical problems affecting seniors, articles on caregiver well-being and managing difficult family dynamics, information on housing options for elders, as well as blogs, discussion forums, and experts in health and eldercare posting answers to visitors' questions. Most of the content is written in-house. The site is supported by venture capital and advertising revenue. For more information on the site and how this may have influenced the sample and survey results, please see methods and discussion sections of the article. |
| | Mandatory/voluntary | Participation in the survey was strictly voluntary. Site visitors could easily close the pop-up invitation and continue using the site if they desired. |
| | Incentives | No incentives to participate were offered. A browser cookie tracked each user, and prevented re-inviting the user to participate in the survey for the duration of the cookie (set to expire after 1000 days). |
| | Time/Date | The survey data was collected between March 2008 and March 2009. |
| | Randomization | No items were randomized or alternated. |
| | Adaptive Questioning | No adaptive questioning was used. |
| | Number of items & number of screens | All five questions could be viewed through just two online screens.<br>The first online screen of the survey included just one question: 1) "How likely is it that you would recommend Caring.com to a friend or relative? Please answer on a scale of 0 to 10, where 10 is "extremely likely" and 0 is "not likely"?" 2161 respondents submitted a response to this question, and hence were advanced to the next screen of the survey.<br><br>The second screen of the survey included the remaining 4 questions: 2) "What were you looking for when you came to the Caring.com website?"; 3) "Did you find what you were looking for?"; 4) "Who are you caring for?"; and 5) "What is your gender?" . On this second screen, participants were not required to answer all questions before clicking "Submit" at the bottom of the screen. |
| | Completeness check | The survey itself did not enforce completeness. The study authors did tabulate incomplete answers for each question, and these are reported in the results. |
| | Review step | Participants were not offered any opportunity to review or change their answers. |

| | | |
|---|---|---|
| Response Rates | Unique site visitor | Unique site visitors are identified by Zoomerang through a browser cookie. To reduce the likelihood of individuals submitting repeatedly, the survey software was set to allow only one response per cookie. |
| | View rate | The number of unique visitors from March 2008 to March 2009, as counted by Google Analytics, was 1,578,309; the number of visits was 1,889,507. The pop-up survey invitation (which technically is a not a pop-up, but rather is a layer of the webpage, hence cannot be blocked by pop-up blockers) was set to appear during 5% of visits, provided the visit lasts over 120 seconds. The number of visits lasting over 120 seconds was not recorded, hence it is not possible to calculate an exact view rate. However, if every visit had lasted 120 seconds, this would correspond to 94,475 views of the survey invitation, so as 9,662 visitors subsequently viewed the first screen of the survey, the view rate is at least 9,662/94,475 = 0.1%. |
| | Participation rate | Of the 9,662 potential participants who clicked the pop-up invitation and viewed the first screen of the survey, 2,161 submitted at least an answer to the first question. This corresponds to a participation rate of 22.4%. |
| | Completion rate | Of the 2,161 respondents who submitted the first survey page, 2145 submitted the second survey page, resulting in a completion rate of 99%. |
| Preventing multiple entries | Cookies used | Cookies were used by Caring.com to uniquely identify users; the cookie can be set by any page on the website, and is programmed to expire after 1000 days. Cookies were also used by Zoomerang to uniquely identify users who agreed to participate in the survey and were redirected to Zoomerang's site. The length of validity of Zoomerang's cookie during the study period is unknown. |
| | IP check | None was used. |
| | Log file analysis | Not used. |
| | Registration | No registration was required for survey participation. |
| Analysis | Handling of incomplete questionnaires | All questionnaires which included submission of at least the first survey page were included in our analysis. |
| | Questionnaires submitted with atypical time stamp | Time stamps were not analysed. |
| | Statistical correction | No statistical technique was attempted to correct for a non-representative sample. |
